# A Review on Development and Applications of Bio-Inspired Superhydrophobic Textiles

**DOI:** 10.3390/ma9110892

**Published:** 2016-11-03

**Authors:** Ishaq Ahmad, Chi-wai Kan

**Affiliations:** Institute of Textiles and Clothing, The Hong Kong Polytechnic University, Hung Hom 00852, Kowloon, Hong Kong, China; ahmadrai621@gmail.com

**Keywords:** superhydrophobic textiles, self-cleaning, oil–water separation, UV-protection, contact angle

## Abstract

Bio-inspired engineering has been envisioned in a wide array of applications. All living bodies on Earth, including animals and plants, have well organized functional systems developed by nature. These naturally designed functional systems inspire scientists and engineers worldwide to mimic the system for practical applications by human beings. Researchers in the academic world and industries have been trying, for hundreds of years, to demonstrate how these natural phenomena could be translated into the real world to save lives, money and time. One of the most fascinating natural phenomena is the resistance of living bodies to contamination by dust and other pollutants, thus termed as self-cleaning phenomenon. This phenomenon has been observed in many plants, animals and insects and is termed as the Lotus Effect. With advancement in research and technology, attention has been given to the exploration of the underlying mechanisms of water repellency and self-cleaning. As a result, various concepts have been developed including Young’s equation, and Wenzel and Cassie–Baxter theories. The more we unravel this process, the more we get access to its implications and applications. A similar pursuit is emphasized in this review to explain the fundamental principles, mechanisms, past experimental approaches and ongoing research in the development of bio-inspired superhydrophobic textiles.

## 1. Introduction

Modern research is offering such forms of bio-engineering which are reliable and revolutionary enough to transform the conditions of our existence. Nature is full of infinite enigmas awaiting an answer and implementation in the best human interest. Among the unbounded natural processes, the self-cleaning phenomenon in living beings captured the attention of scientists and researchers owing to its vast applications in discrete fields. This phenomenon was first discovered by two German botanists, Barthlott and Neinhuis, in lotus plant leaves which get washed off by rolling water droplets on the surfaces. They noticed that the waxy surface structure of lotus leaves has low adhesion of water droplets which renders it non-wettable [1,2]. The lotus leaf has been a sign of sacred purity. The verse by a Chinese poet “the lotus and leaves all over the pond, and breeze blows beads roll down” describes the self-cleaning phenomenon of lotus leaves as water drops falling them roll off and wash dirt from the leaves to make them clean. This self-cleaning effect is worldly known as the “lotus effect” [3,4]. Studies have revealed that the lotus effect is not unique to lotus leaves; some other plants and insects have the natural tendency to get cleaned by rolling water drops on their surfaces, such as rice leaves, fish scales, butterfly wings, salviniamolesta, mosquito eyes and shark skin [5,6,7,8,9,10].

This lotus effect is directly related to the interaction of a water droplet with a solid surface. A considerable number of studies have been conducted to explain the interacting behavior of water droplets on solid surfaces and, in this regard, various theories have been established including Young’s equation, and Wenzel and Cassie–Baxter theories [11,12,13]. However, a complete description has not been given yet to explain the exact mechanism of wetting of surfaces with water droplets and still this is a hot topic of research in academia and industries. In this review, a concise attempt has been made to discuss recent research advances in self-cleaning of hydrophobic surfaces with regard to the basic theories of wetting, measurement of contact angles, factors affecting the wetting process and future prospects in this cutting-edge field of study.

### 1.1. Wetting Process

**Figure 1:**
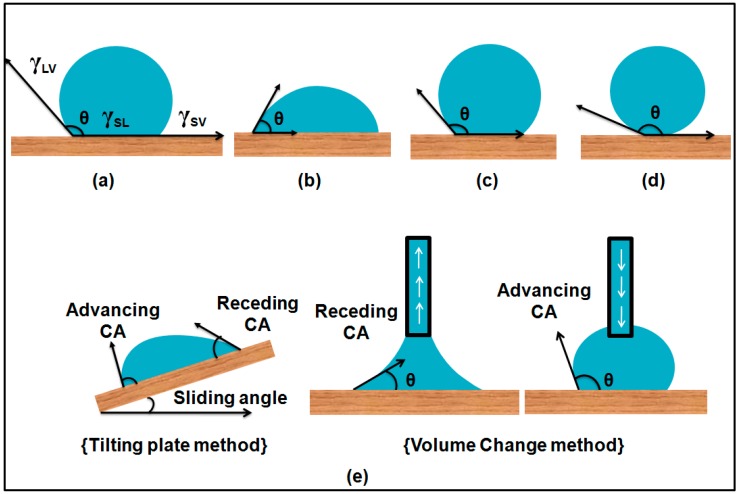
Schematic definition of hydrophilicity, hydrophobicity and superhydrophobicity: (**a**) Interfacial tensions of all three phases that co-exist and static contact angle; (**b**) Hydrophilic surface contact angle(θ) <90°; (**c**) Hydrophobic surface contact angle(θ) >90°; (**d**) Superhydrophobic surface contact angle(θ) >150° and (**e**) Dynamic contact angles for measurement of contact angle hysteresis.

Wetting of a solid surface is termed as the contact of a liquid with the solid surface as a result of intermolecular interactions. The balance between adhesive and cohesive forces determines the extent of wettability of the solid surfaces. Adhesive forces between the solid surface and liquid droplets result in spreading on the solid surface while asymmetric cohesive forces among the liquid molecules cause surface tension and thus reduce the area of interaction between liquid and solid surface and make the droplets spherical in shape. When a liquid drop comes into contact with a solid surface, a contact angle θ (CA) between liquid–air interface and the solid–liquid interface is formed as shown in the Figure 1.

In the wetting process, an area of solid–air interface is replaced by an equivalent area of solid–liquid interface along with some extension of the liquid–air interface. These surface relations depend on the conditions of wetting, nature of the liquid and surface properties of the solids. Furthermore, these surface relations are associated with the energy content of each interface. As wetting is a thermodynamic process, the total energy content of each interface changes after wetting. The sign of the change in surface energy involved determines whether the wetting process will occur spontaneously or non-spontaneously. These surface energy changes also help to determine the rate of the wetting process, how far it proceeds against the external forces that may resist to the wetting and how much external force will be needed to overcome the resistance to wetting [11].

### 1.2. Young’s Equation and Contact Angles

In order to explain the behavior of water droplets on a solid surface, Thomas Young, in the 19th century, proposed a mathematical equation which explicitly relates the contact angle θ (Young Contact Angle) to interfacial energies (surface tensions). In detail, when a liquid drop comes into contact with an ideal flat, rigid, homogeneous and inert surface, three interfaces—solid–liquid (SL), solid–gas (SV) and liquid–gas (LV) co-exist with a contact line formed between these interfaces. Interfacial energies play a major role in determining the contact angle (θ) of a liquid drop with the solid surface. The equation was derived by balancing the forces (surface tensions) on the contact line of the liquid drop and solid surface (x-axis):

ΣFx = γ_SV_ − γ_SL_ − γ_LV_cosθ = 0 (1)

γ_LV_cosθ = γ_SV_ − γ_SL_ (2)

cosθ = (γ_SV_ − γ_SL_)/γ_LV_ (3)

where γ is interfacial tension expressed in terms of energy per unit area of the interface and θ is the contact angle between the liquid droplet and solid surface respectively, as shown in the Figure 1a.

According to Young’s equation, the contact angle θ quantifies the wettability of a solid surface by liquid droplets. For a smooth surface, Young’s contact angle increases when γ_SV_ decreases or γ_LV_ increases. If the Young’s contact angle is less than 90°, the wetted surface will be energetically more favorable than the dry solid surface and is thus said to be hydrophilic. If the Young’s contact angle is greater than 90°, the dry solid surface will be energetically more stable and the surface is termed as hydrophobic. If the contact angle is greater than 150°, the surface is termed superhydrophobic [14,15]. For oils, the corresponding terms describing favorable and non-favorable conditions are oleophilic, oleophobic and superoleophobic; for other non-aqueous liquids, lyophilic and lyophobic terms are used, while for all kinds of liquids, omniphilic and omniphobic words are commonly used.

### 1.3. Wenzel Model

However, an ideal flat, rigid, homogeneous and inert surface does not exist in nature and real surfaces vary in their surface properties. Every surface has some proportion of roughness at molecular or atomic levels, thus Young’s equation cannot be used to explain the exact mechanism of wetting. Wenzel then developed an equation to explain the effect of surface roughness and surface energies on the contact angle [11,16]. Wenzel’s equation is given as:

cosθ* = rcosθ
(4)

where θ* is the Wenzel contact angle and r is surface roughness of the solid surface. Roughness is defined as “ratio of the actual surface to that of a smooth surface having the same geometrical shape and dimensions”.

Roughness (r) = actual surface/geometrical surface(5)

**Figure 2:**
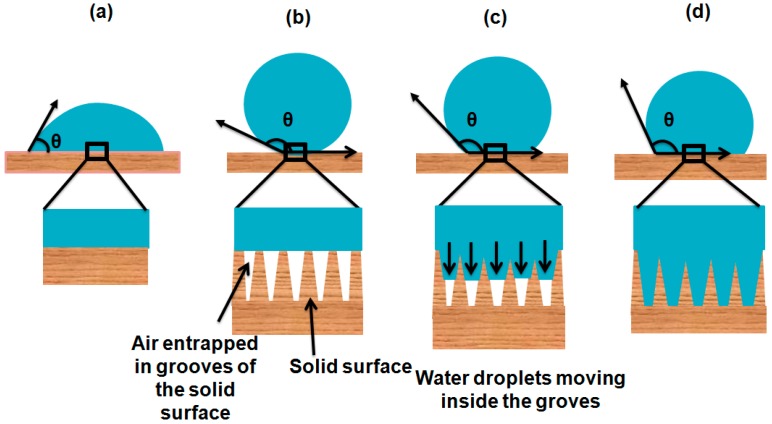
Schematic illustration of theories of superhydrophobicity: (**a**) Young’s state, droplet sits on the smooth surface; (**b**) Cassie–Baxter state, water droplet partially standing on solid surface and entrapped air; (**c**) Intermediate state of Cassie-Baxter and Wenzel state, water droplet moving inside the grooves; (**d**) Wenzel state, water droplet wetting the solid surface completely.

According to the Wenzel model, a liquid drop completely wets the solid surface by entering into all the anfractuosities caused by the surface roughness and solid–liquid interface is increased as shown in Figure 2d. From Wenzel’s equation, superhydrophobicity and superhydrophilicity can be predicted. Surface roughness decreases the hydrophobic character of an intrinsically hydrophilic surface (<90°) and increases the hydrophobic properties of an intrinsically hydrophobic surface (θ >90°) θ [17,18,19].

### 1.4. Cassie–Baxter Model

The Wenzel model is only applicable to a homogeneous regime in which a liquid droplet fully penetrates into the grooves of the surface. Later, it was proposed by Cassie and Baxter that some air is entrapped in the grooves of the solid surface and liquid droplets sit on the top of protrusions, thus this surface is termed as a heterogeneous regime [12]. The fraction of the solid surface that directly contacts with the liquid droplets is denoted as Φs. Cassie and Baxter developed an equation to highlight the effect of the actually wetted area of the solid surface on the contact angle given as:

cosθ* = −1 + Φs(cosθ + 1)(6)

where θ* is the apparent contact angle. This equation shows that roughness alone is not enough to explain the contact angle of the liquid drop with the solid surface but it is the total area that is directly in contact with the solid surface which determines the contact angle, thus total wettability of the solid surface can be calculated. To this point, it is suggested that Young’s equation gives the basic idea for wetting phenomena on an ideal, smooth and non-reactive surface, Wenzel’s equation applies to a homogeneously wetted surface and the Cassie–Baxter equation is only applicable to a heterogeneous state in which air is entrapped underneath the liquid droplet as shown in the Figure 2b. However, the wetting phenomenon is not as simple as it seems in these theories because these three models do not explain the stability of the Cassie–Baxter state, transitions from the Cassie–Baxter state to the Wenzel state, the effect of size of water drops and the nature of solid surfaces. To explain it in more detail, further studies have been conducted [20,21,22].

### 1.5. Dynamic Contact Angles and Contact Angle Hysteresis

Three thermodynamic parameters, γ_LV_, γ_SV_, and γ_SL_ in Young’s equation are used to determine a single static contact angle θ. In reality, a liquid droplet undergoes many metastable states on a solid surface and contact angles measured for these states are usually not equal to Young’s contact angle θ. Hence, wetting behavior of a solid surface cannot be explained by measuring a single static contact angle. When contact lines of all the three phases are in motion, a dynamic contact angle is formed with the solid surface. This dynamic contact angle varies either by the contact motion with the surface or by increasing or reducing the size of the liquid droplet. The variation is in between a range of minimum and maximum values. The maximum contact angle of this range is usually termed as the advancing contact angle (θa) and the minimum angle is usually termed as the receding contact angle (θr) as shown in Figure 1e, but this association of θa and θr as maximum and minimum are under study, so one should be cautious when assigning these angles [23]. The difference between the advancing contact angle and the receding contact angle is called the contact angle hysteresis. The contact angle hysteresis value depicts whether the surface is wettable or non-wettable. For an aqueous system, a higher value of H is associated with hydrophilic surfaces (wettable) and a low value approaching zero is associated with superhydrophobic surfaces (non-wettable) [14,24].

Contact angle hysteresis (H) = advancing contact angle (θa) − receding contact angle (θr)(7)

### 1.6. Importance of Contact Angle Hysteresis

Measurement of dynamic contact angles and contact angle hysteresis is very important for complete understanding of the mechanism of the wetting phenomenon. Its significance has been studied by many researchers. Tsujii and his team prepared fractal surfaces from alkylketene dimer (AKD) to study the contact angles and water repellency of the surfaces. They observed the maximum contact angle of 174° and they suggested that maximum contact angle of 180° can be achieved if liquid droplets have no adsorption on the solid surfaces [25,26]. Their study opened new doors for researchers to prepare superhydrophobic materials for practical applications having extremely high contact angles, even approaching up to 180° [27,28,29,30,31,32,33]. However, in these studies they did not give the complete measurements of contact angle hysteresis. As mentioned earlier, a single maximum value of contact angle is not adequate to explain the wetting phenomenon and get the stable superhydrophobic surfaces. To describe the stability of the superhydrophobic surface, both static contact angle and dynamic contact angles are necessary to be measured. To get a very stable superhydrophobic surface, the static contact angle should be very high and contact angle hysteresis should be as low, approaching zero. Contact angle hysteresis is normally lower in the Cassie–Baxter state which makes it more stable than the Wenzel state [11]. If the contact angle hysteresis difference is higher between the Wenzel and Cassie–Baxter states, then there might be a transition from the Wenzel to the Cassie–Baxter state making the wetting metastable [34,35]. Moreover, both the Cassie–Baxter and Wenzel states are extreme states and when external pressure is applied on the Cassie–Baxter surface, a transition will occur from the Cassie–Baxter state to the Wenzel state while passing through the various intermediate states shown in Figure 2c.

### 1.7. Stability of Cassie–Baxter State

Obtaining superhydrophobic surfaces with high stability requires surfaces which can support high external pressure without transitioning from the Cassie–Baxter state to the Wenzel state. These stable surfaces are termed as robust surfaces. Stability or robustness of the the Cassie–Baxter state is highly dependent on the substrate surface morphologies. The surface treatment of substrates at micro and nano-scale level may increase its roughness and robustness. The major parameter that enhances the robustness of the surface is the presence of re-entrant topographies.

### 1.8. Re-Entrant Topographies

The presence of re-entrant structures on the surface stabilizes the Cassie–Baxter states for robust superhydrophobic surface. Re-entrant topographies have been reported in two types of patterns i.e., random and regular patterns. Re-entrant structures with random patterns have been reported by using nano-particles, nano-wires and nano-pores prepared by simple cost effective methods such as chemical synthesis, electrospinning and candle soot depositions [36,37,38]. However, it has been found that, in random patterns, it is difficult to control the uniform surface morphologies on large areas. Re-entrant structures with regular patterns have been prepared in the form of T-shaped micropillars [39,40,41], nanonails [42], microhoodoos [43], textile fibers [44,45] and inverse trapezoids [46,47]. These regular re-entrant topographies have been developed by chemical etching and microinjection compression molding (µ-ICM).

## 2. Measurement of Contact Angle

The wetting process of a solid surface is a complex phenomenon and detailed studies of how liquid droplets interact with the solid surfaces and of how wetting occurs are under research. Exact measurement of Young’s contact angle and dynamic contact angles is of great importance to explain the wetting mechanism in detail. With the development of theories of the wetting process, the tools and techniques used to measure the contact angle of liquid drops with solid surfaces are also being developed from very basic to some advanced techniques.

### 2.1. Methods Used for Sessile Drops on Solid Surfaces

According to Young’s theory, when a sessile liquid droplet comes into contact with an ideal flat, rigid, homogeneous and inert surface, it makes a contact angle with the surface. In the early stages of contact angle measurement, some basic techniques were used.

#### 2.1.1. Direct Goniometer Method

The direct goniometric method is the most commonly used method for the measurement of liquid contact angles on solid surfaces. For the first time, the contact angle of liquids on solid surfaces was measured by Bigelow and his co-workers in 1946 during the study of oleophobic and hydrophobic films on solid surfaces. They developed a simple set up and named it telescope-goniometer [48]. The first commercial instrument for the measurement of contact angles in the world was manufactured by a ramé-hart company. This instrument was designed by William Zisman in the early 1960s. The instrument consists of four basic components: (1) a horizontal surface to mount a liquid or solid sample; (2) a micropipette to form a liquid droplet on the surface; (3) an illuminating source; and (4) a telescope assembled with a protractor eyepiece.

The basic working principle of this instrument centers on the assignment of a tangent line to the sessile drop at the contact point with the solid surface. In detail, a sessile droplet of a liquid of known boiling point and density is placed on the horizontal surface by an equipped micropipette. Then, the tangent line is aligned to the contact line of the droplet with the solid surface and the contact angle reading is noted with the help of a protractor eyepiece. Modern systems now fit a curve to the contour of a video image and employ computer software to determine the contact angle.

Advantages of the method:

The direct telescope-goniometric method is very simple to use and it has the following advantages:

- Only a few microliters of the liquid sample are required.
- Surface contact angle of very small substrate surface i.e., only few square millimeters can be measured.
- An accuracy of direct contact angle of approximately ±2° is generally achieved by using the telescope-goniometric method [49,50].
- With the passage of time, modifications have been made in the method to get better accuracy and precision i.e., (1) integration of a camera to take images for future analysis and presentation; (2) use of high magnification lenses to get an in detail examination of the intersection profiles of contact lines of all the phases in contact; and (3) assembly of a motor driven micro syringe to control the volume of the liquid droplet [23,51,52].
- Zisman and his co-workers used a very simple method for the measurement of contact angle of sessile drops on solid surfaces by using a platinum wire. In this method an 8 cm long platinum wire of about 0.05–0.1 mm diameter is cleaned by heating with a Bunsen burner. This cleaned wire tip is dipped into the liquid and gently removed from the liquid to get a suspended drop on the tip of the platinum wire. This pendent drop is smoothly brought into contact with the solid surface, forming a sessile drop on the surface and the wire is gently removed from the drop. Furthermore, the contact angle was noted with the help of telescope-goniometer. Reproducibility for this method is claimed to be of ±2°.

Precautions of using this method:

While measuring the accurate contact angle using this method, the following precautions are very important:

- To avoid inconsistency and uncertainty in measuring the contact angle, it is suggested to establish general guidelines for the users.
- A background light as an illumination source is used, but it is necessary to use a light source of specific intensity to avoid the undesired heating and evaporation of the droplet.
- To measure the dynamic contact angle, a micro syringe with a stainless steel needle is used to increase or decrease the volume of the liquid droplet. During this process, the needle must remain in the droplet to avoid unnecessary vibrations.
- If the substrate is of a large area, it is important to measure the contact angles at different points to get an average value representing the whole surface of the substrate.
- An enclosed chamber should be used for all the measurements to prohibit airborne contamination and vapor pressure equilibrium of the liquid under study must be obtained for volatile liquids.
- The droplet may have unsymmetrical attachment with the surface; it is appropriate to measure the contact angles on both sides of the liquid droplet to get more accuracy.

Limitations of the method:

Although the direct telescope-goniometric method has advantages because of its simplicity and ease of use, it has some shortcomings which reduce its use for further insights into the wetting processes and dynamic contact angle measurements. Some of the drawbacks are shortlisted here:

- The accuracy and reproducibility of the contact angle measurements rely on the consistency of the user in assigning the tangent line. When measurements are taken by multiple users, a significant error and inconsistency might occur.
- The direct telescope-goniometric method has another serious limitation. On superhydrophilic surfaces, water droplets are almost flat and it is very difficult to assign a tangent line, thus accurate contact angles below 20° cannot be measured by this method.

#### 2.1.2. Conventional Tilting Plate Method

**Figure 3:**
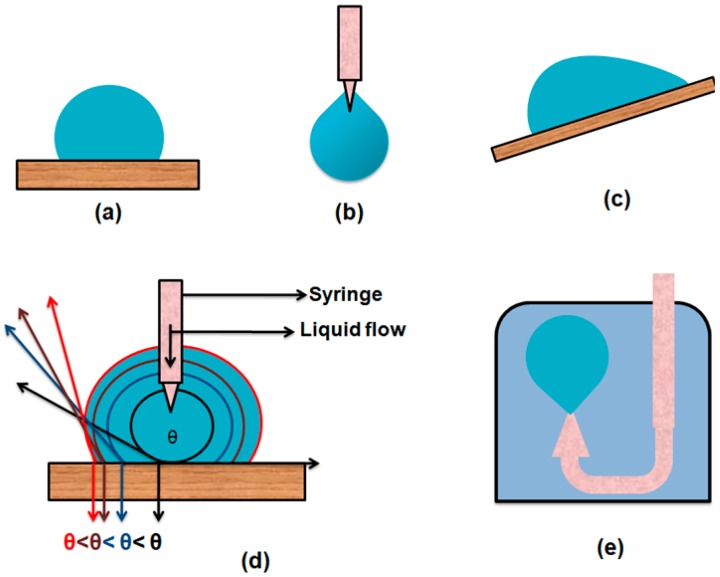
Schematic illustration of types of drops: (**a**) Sessile drop on a flat and rigid surface; (**b**) Pendant drop; (**c**) Tilting drop for measurement of dynamic contact angles; (**d**) Change in dynamic contact angle by changing the drop volume; (**e**) Reverse pendant drop for measurement of interfacial tensions.

To study dynamic contact angles and contact angle hysteresis, McDougall and Ockrent [53] modified the sessile drop method (Figure 3). In this approach, a sessile drop is formed on the solid surface and the surface is inclined slowly until the drop rolls off or slides on the surface. At the time of sliding of the drop, it forms two angles with the solid surface. One at the lower side of surface termed as the advancing contact angle and one on the upper side of the surface which is termed as the receding contact angle as shown in Figure 1e. Extrand and Kumagai used this method to study the dynamic contact angles and contact angle hysteresis for liquid drops on various solid surfaces including silicon wafers, polymer surfaces and elastomeric surfaces [54,55].

Limitations of the method:

The dynamic contact angle values obtained by this method are not reliable as studies have shown that the values of the sliding angle, advancing contact angle and receding contact angle may vary to a noticeable extent depending on the nature of solid surface, local surface energies, nature of liquid drops, interplay with drop volume, drop shape and the point of placement of liquid drop on the tilting plate [56,57,58].

#### 2.1.3. Tangentometric Technique

This approach was used by Phillips and McIntyre in their studies. In this method, the drop profile on the solid surface is analyzed by a setup named as tangentometer-protractor device. This system consists of a mirror placed underneath the baseline of the water drop and the reflected image of the drop is taken. Then the mirror is placed vertical to the image at the drop tip and is rotated to get a smooth curvature and this curvature is matched with that of the reflected image and the contact angle is observed by the protractor attached with the mirror [59]. However, this method is not useful as it has uncertainty in the practice of users.

### 2.2. Methods Used for the Measurement of Contact Angles on Solid Surfaces with Different Geometries

In the methods above, the most common techniques used for the contact angle measurements have been discussed in which the liquid drops are mostly sessile. In this section, the techniques and instruments used for the contact angle measurements for the solid surfaces with different surface arrangements are discussed.

#### 2.2.1. Captive Bubble Method

This method was first introduced by Taggart and coworkers [60]. With the passage of time and several modifications, it is now most commonly called the “captive bubble method”. In this method, the solid sample is immersed in the liquid under study and then an air bubble is introduced beneath the solid surface. The air bubble makes a contact angle inside the liquid which can be directly measured.

Advantages of this method:

This method offers following advantages:

- This method ensures that the solid surface is immersed in the saturated environment.
- Minimum air born contamination on the solid–air interface.
- Easy to control the temperature of the system under study which makes it easier to study the effect of temperature on contact angles.
- For clean polymeric surfaces, the contact angle measurements are in good agreement with that of the sessile drop methods [61].

Limitations of this method:

This method suffers with following limitations:

- It requires large amount of liquid as compared to the sessile drop methods.
- It is not easy to measure the contact angle of solid surfaces that swell when coming into contact with the liquid or substrates with soluble thin films present.

#### 2.2.2. Tilting Plate Method

The simple titling plate method used for measurement of the contact angle was developed by Adam and Jessop. In this technique, a simple solid plate is used. The plate is gripped from one end and the other end is slowly immersed in the test liquid so that a meniscus forms on both sides of the plate. Then the plate is tilted to one side until one of the meniscuses becomes flat with the liquid surface. At this stage, the contact angle formed between the tilted solid plate and the flat liquid surface is measured. They reported an error of ±5°attributed to the contamination in the liquid. To control the movement of the solid plate and avoid surface contamination, glass barriers are used on the surface of the liquid. This method has been improved by equiping the system with a scanning laser beam and thermocapillary (TC) responsive tool in order to observe the moving contact line and static curvature of the liquid meniscus respectively. The major advantage of this method is easy measurement of the low contact angle [23,62,63].

Limitations of the method:

Major limitations reported in this method are:

- When the solid plate is immersed in the test liquid, a disturbance in the liquid appears that may affect the exact measurement of the contact angle.
- Observation of the moving solid plate in the test liquid when the meniscus on one side becomes parralal to the plate requires highly skilled persons for accurate measurement of the contact angles [23].

#### 2.2.3. Wilhelmy Balance Method

**Figure 4:**
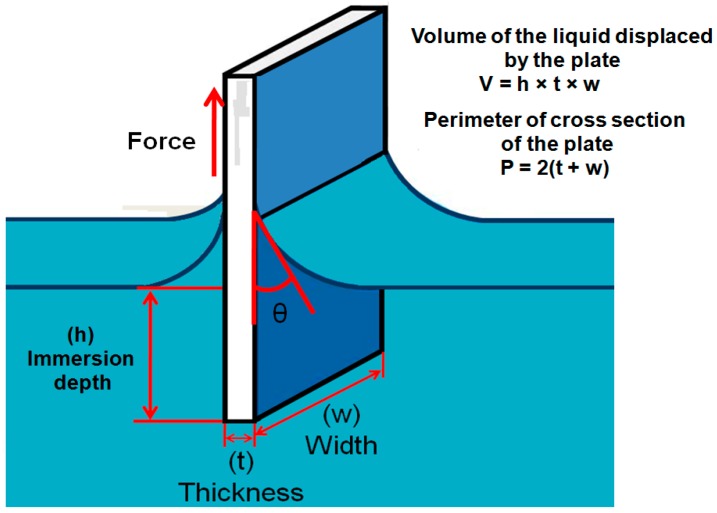
Schematic diagram of the Wilhelmy balance method.

In this technique, a vertically oriented, solid thin plate suspended from a micro balance is brought into contact with the test liquid (Figure 4). The contact angle can be determined indirectly from the force that applies on the solid plate when it penetrates the surface of the liquid and produces a weight change of the system. The force change on the balance that is associated with the combination of wetting force and buoyancy is noted. The force change on balance is shown in (8):

F = mg + γ_L_Pcosθ − Vρg (8)

where mg is weight of the solid plate, γ_L_ is surface tension of the liquid, P is perimeter of the cross section of the solid plate which is in contact with the liquid, θ is the contact angle, ρ is density of the liquid, V is volume of the liquid displaced by the solid plate and g is gravity acceleration. In this equation γ_L_Pcosθ represents the wetting force and Vρg represents the buoyancy. When surface tension of the liquid and perimeter of the cross section of the solid plate are known, the contact angle can be measured using this equation [64].

**Figure 5:**
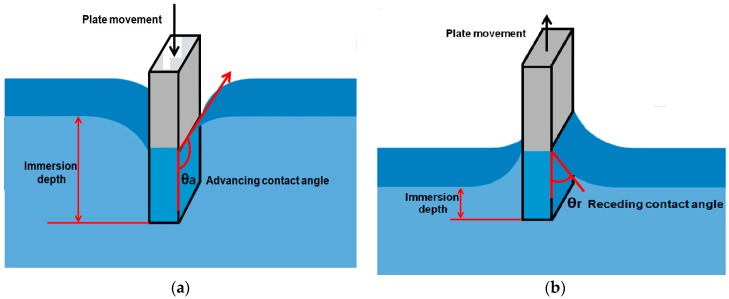
(**a**) Schematic diagram of the Wilhelmy balance method to measure the advancing contact angle; (**b**) Schematic diagram of the Wilhelmy balance method to measure the advancing and receding contact angles.

Advantages of the method:

This method has several advantages:

- The contact angle is indirectly calculated from the measurement of weight force and length which can be measured with more accuracy that direct observation of the contact angle.
- The force can be measured from any depth in the liquid which is always an average value.
- Measurement of the contact angle by this method is more accurate and the advancing and receding angles can be measured by this approach as shown in the Figure 5.

Limitations of the method:

Along with the advantages, it also has some drawbacks:

- From this method, surface properties of the solid-like homogeneity and heterogeneity cannot be predicted.
- In this method, the solid sample must be of a known cross sectional area, making it limited for only rods, plates and fibers.
- It can be difficult to measure the precise perimeter of the wetted area of the solid sample.
- Along with the geometry, the solid sample must be the same in composition and topography in all sides which is difficult to obtain if one wants to study the films of anisotropic systems.

#### 2.2.4. Capilary Rise on a Vertical Plate

**Figure 6:**
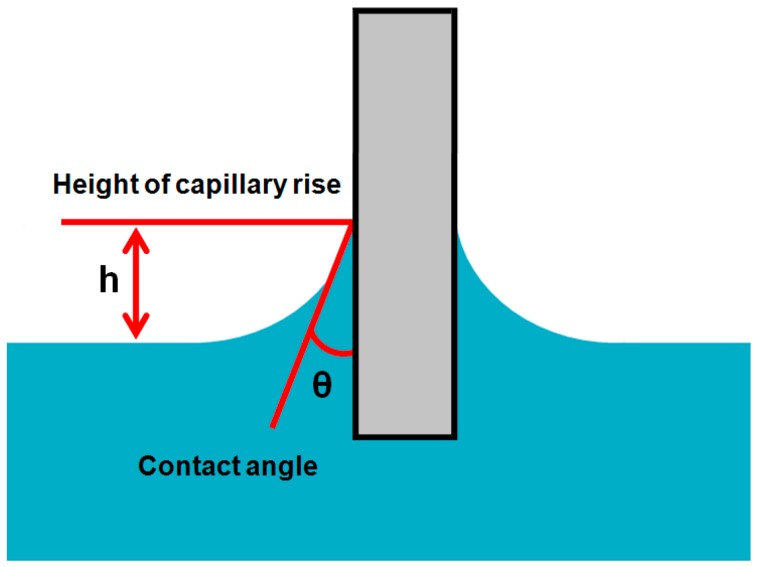
Contact angle measurement by capillary rise on a vertical plate.

This approach is also applied for indirect measurement of the contact angle. In this method, an about 2 cm wide plate is immersed in the liquid of known surface tension as shown in the Figure 6. Liquid rises due to the capillary effect on the plate surface. The rise of the liquid by capillary action is measured in terms height (h) at a fixed immersion depth. The contact angle can be measured from this height of cappilary rise by using the modified Laplace equation [65].

Sinθ = 1 − ρgh^2^/2γ_L_ (9)

where ρ is density of the liquid, g is the gravity acceleration, h is height of cappilary rise on the solid plate and γ_L_ is surface tension of the test liquid. This method is more suitable for the temperature dependent contact angles [66,67]. Budziak, Neumann and Kwok et al. [68,69] automated this method for direct measurement of contact angles. Also, in some studies, the above equations have been combined by using the sin^2^θ + cos^2^θ = 1 relation to get the surface tension and contact angles at the same time [70,71]. Precise observation of h plays a key role in the exact measurement of the contact angle.

#### 2.2.5. Capillary Rise in a Vertical Tube

**Figure 7:**
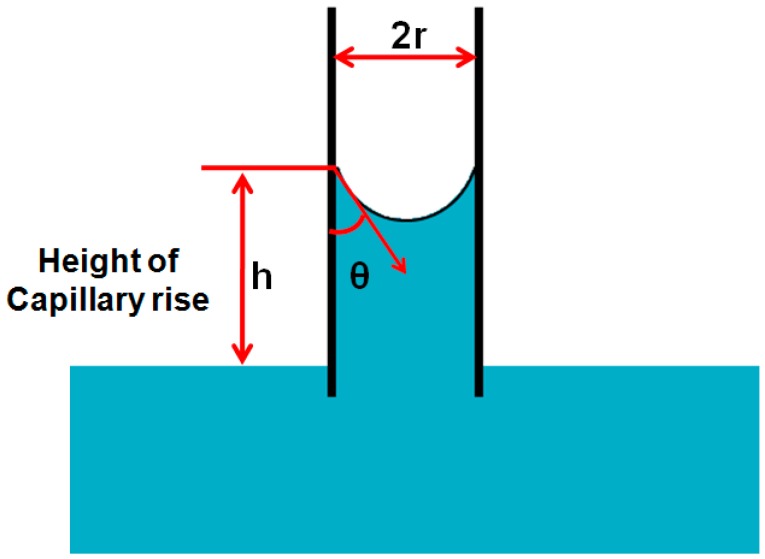
Measurement of the contact angle by the capillary tube method.

A cappilary tube is used in this approach in order to obtain the contact angle. The capillary tube of known radius is placed in the test liquid with known density and surface tension. The liquid rises with capillary action and makes a minescus with walls of the tube. The height of cappilary rise in the tube is measured and the contact angle is measured by using Jurin’s law.

h = 2γ_L_cosθ/ρgr (10)

where ρ is density of the liquid, g is the gravity acceleration, h is height of cappilary rise in the tube, r is radius of the tube and γ_L_ is surface tension of the test liquid. The schamtic diagram of this method is given in Figure 7.

#### 2.2.6. Measurement of Contact Angle on Fibers

In various studies, researchers have tried to measure the contact angle directly on the individual fibers [72]. In this method, the individual fibers are suspended horizontally under a microscope and the goniometer eyepiece is used to note the contact angle of the liquid drops present on the surface of the fibers. Approximate dynamic contact angles can be measured by rotating the fiber longitudinally. In every method, modifications have been made with the passage of time to get better results in terms of accuracy and operating procedures. To get better results of contact angle measurements by this method, Bascom and Romans [73] placed the liquid drop in a small platinum ring and passed a glass fiber through it vertically. Dynamic contact angles were noted while passing the glass fiber from the center of the drop with the help of a microscope. This method has made it easy for many researchers to observe the effect of diameter of individual fibers on the contact angles. It has been reported in some articles that, if the diameter of the liquid drop is greater than that of the fiber, misleading results are obtained as drop curvatures and weight of the drop disturb the drop profile at the intersection of the drop and fibers [74,75,76].

## 3. Theories for Analysis of Drop Profile

As discussed in various previous sections, properties such as drop shape, drop size, drop formation, surface tension and surface morphologies are major parameters that determine the contact angle. Along with the method development to get the contact angle, it is also important to develop the theoretical models that completely describe the contact angles measured experimentally from the drop shape and explain the individual effects of all these parameters on the contact angle. In the early beginning of this research area, it was found that interfacial and gravitational forces have the combined effect on the shape of drop. Surface tension reduces the surface area and makes the drop spherical while gravitational forces either make the pendant drop sag or flatten the sessile drop. To completely analyze the drop profile, it is very important to consider the force balance of these parameters. This force balance, including the Laplace equation, gives a way to measure surface tension and the drop shape precisely [77]. Two basic methods that have been widely used to analyze the drop shape are (1) θ/2 method; and (2) Axisymmetric Drop Shape Analysis (ADSA) [78,79,80].

### 3.1. Drop Shape Analysis by θ/2 Method

**Figure 8:**
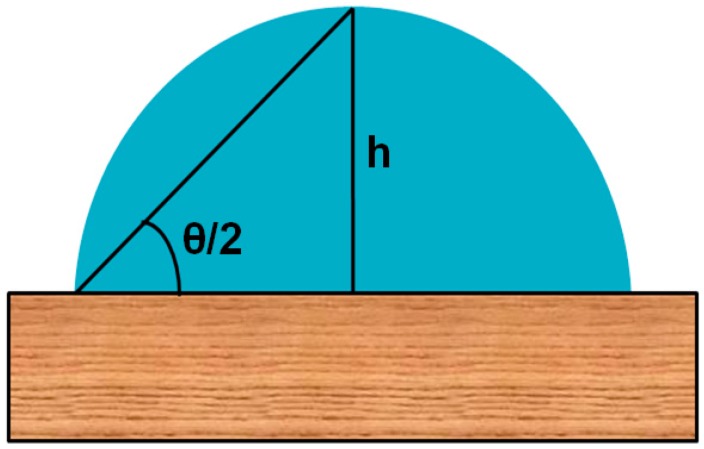
Schematic illustration of the drop shape analysis by θ/2.

This method has been mostly used for the analysis of sessile drops on a flat surface (Figure 8). In this method, the drop is considered as a part of a sphere and the geometrical contact angle is obtained by observing the diameter of the drop and height of the apex from the solid surface. A simple equation that has been used to calculate the contact angle from the height of the apex and the diameter of the drop is given as:

θ/2 = tan^−1^(h/d) (11)

where h is the height of the apex and the d is diameter of the base of the drop. This method is applicable only when the drop is spheroidal, even when the drop size is very small. If the drop size is large and the drop shape is not spherical due to external forces, this method is of no use. Bashforth and Adams formulated a table of surface tension values generated from the Laplace equation manually for various drop profiles. Later, this table was extended for the pendent drops as well, but with the development of digital computers, tremendous developments in theoretical models have been made to analyze the drop profile [81,82,83].

### 3.2. Axisymmetric Drop Shape Analysis (ADSA)

In last few decades, significant developments have been made in the design of hardware and computational theories that led researchers to explain the drop profile and analyze the surface science. Axisymmetric Drop Shape Analysis (ADSA) is the most widely used technique for analysis of the drop profile and it was first developed by Rotenberg and coworkers [84,85]. Later, various modifications have been made in this model to provide better reproducibility and precision. The ADSA method has improved the precision of contact angle values by an order of magnitude as compared to direct tangent measurements. The ADSA method uses a basic principle in which the theoretical model is developed that explains the drop profile obtained from the experimental image. The analysis gives values of contact angle, drop shape, drop volume, surface tension and wetted surface area. This ADSA method has many versions, improved at various stages of the developments i.e., (1) first model termed as ADSA-P [86]; (2) second model named as ADSA-S [80]; (3) third model termed as ADSA-D [87,88]; and (4) fourth model termed as theoretical image fitting analysis (TIFA). Many studies have been reported in the development of these models [89,90,91].

## 4. Superhydrophobic Surfaces in Plants

Naturally designed functional systems in living bodies inspire scientists and engineers worldwide to mimic the system for practical applications by human beings. One of these natural phenomena is the water repellent self-cleaning surfaces observed in many plants and animals [92]. Lotus plant leaves were first to be observed, having hydrophobic surfaces [1]. The Lotus leaves are not only water repellent but also have low adhesion with particulate contamination, thus demonstrating the self cleaning phenomenon perfectly. The reported contact angle for lotus plant leaves is 160° with a contact angle hysteresis of 4. The hierarchical surface of lotus leaves is composed of convex cells and three dimensional waxy tubules. Air is entrapped in the convex cell grooves resulting in the Cassie–Baxter state in which the water droplet rests mostly on the entrapped air and has minimum contact with the leaf structure which makes it superhydrophobic [2]. Furthermore, there are some other plants which have a water repellent hairy surfaces on the leaves. Some of these are the leaves Lady’s Mantle and water ferns Salvinia. Water repellency of these surfaces results from the presence of waxy crystals present in the micro surface protrusions [93].

Micro scale elliptic protrusions and nano scale pins on the surface of taro plant (*Colocasia esculenta*) leaves make hierarchical morphology which imparts superhydrophobic self-cleaning properties to it [88]. A similar kind of binary surface structures (micro and nano-scaled) have been reported in rice and Indian canna (*Canna generalis bailey*) leaves. Wilhelm Barthlott and coworkers demonstrated that the lotus leaves, taro leaves and Indian canna leaves have tubular-platelet type waxy crystals. These waxy protrusions are the most important and prominent factors in making the surface structures water repellent [94,95].

## 5. Superhydrophobic Surfaces in Animals and Insects

Roughness and hierarchical morphology based superhydrophobicity is present not only in plant surfaces; some animals and insects also have micro and nano-scaled surfaces topographies with needle shaped and overlapping edges such as protrusions [6,96,97,98]. These surface topographies result in low adhesion, low drag and superhydrophobicity. The non-wetting superhydrophobic surface of water strider’s (*Gerris remigis*) legs prevents it from shrinking and enables it to move on the water surface more quickly. It has been mentioned that this remarkable superhydrophobicity is due to the wax secreted by the surface of the legs. The reported force of repulsion caused by surface tension of this waxy layer on a single leg is 152 dynes [99] and the water striders can walk on the water surface because their weight is low. Superhydrophobicity and low adhesion morphologies of butterfly wings are due to the regular arrangement of overlapping roof tiles-like edges. Moreover, shark skin exhibits antifouling, self cleaning and low drag which enable it to move fast in water. The shark skin surface is considered to have micro-structured riblets which reduce the adhesion and drag to prevent fouling [6].

However, it is important to mention here that the exact mechanism and factors that make the surfaces superhydrophobic are still under study, making it a field of research. Not all the surfaces having hierarchical surface topographies exhibit superhydrophobicity. For example, the hierarchical morphology found on the gecko foot surfaces has hundreds of micron and sub-micron keratinous hairs and spatula, but it still lacks the superhydrophobicity. Some other animals and plants such as rose petals have both high apparent contact angles and high hysteresis, which is very interesting for other applications, such as in water harvesting.

**Figure 9:**
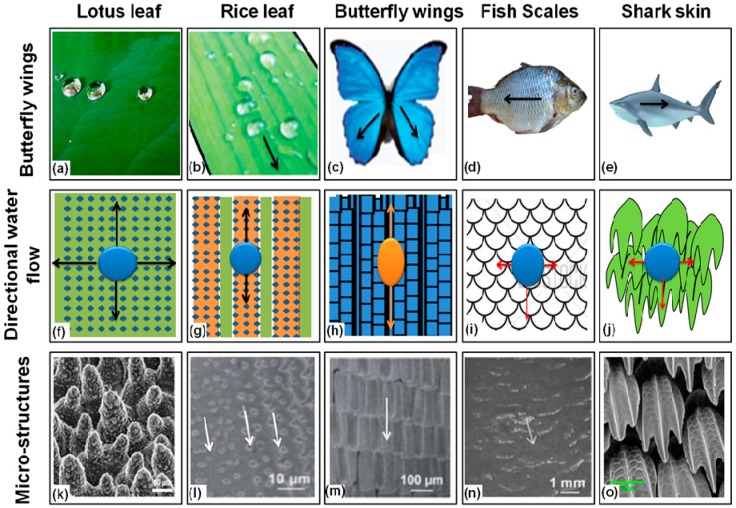
Schematic illustration of water flow on natural superhydrophobic surfaces. Images (**a**–**e**) represent the flow of water droplet on the natural surfaces of lotus, images (**f**–**j**) represent the schematic microstructure of natural surfaces and images (**k**–**o**) are micrographs of the natural surfaces. Images (**k**–**o**) reprinted from the [6]. Copyrights, The Royal Society of Chemistry 2012.

Along with the surface morphology, surface chemistry and pattern of surface structures play a major role in determining the superhydrophobicity and superhydrophilicity of surfaces. Water flow on the animal and plant superhydrophobic surfaces is illustrated in the Figure 9. Physical properties of natural superhydrophobic systems are summarized in the Table 1.

## 6. Superhydrophobic Textiles

Human beings have a highly developed anatomy and physiology but from the beginning of their evolution they found inadequate protection of their bodies from a variety of unfavorable and harsh environmental conditions. To protect their bodies they used additional coverings in the form of clothing on their body parts in different manifestations. Textile materials have been used throughout the human history for clothing. The term “textiles” has been derived from the Latin word “texere” which has the meaning of “to weave”. Earlier, this term was used only for woven fabrics but now-a-days textile fabrics have been manufactured via many processes including weaving, knitting and other technologies. The weaving process involves the interlacement of many perpendicular threads or yarns over each other while in the knitting process a single unbroken strand of yarn goes on intertwining into loops forming the fabric one row at the time. Weaving and knitting patterns can be changed to develop a variety of textile fabrics with many advanced properties. Non-woven fabrics can also be developed by the entanglement of the fibers. In other words the term textiles may be defined as “a thin, soft and flexible sheet of fabric made up of natural polymeric or synthetic fibers with enough strength and tear resistance to be used for clothing and other protective functions” [100].

Since the last century, science and technology have made revolutionary developments at an extraordinary rate in many aspects of textiles and clothing industry. Tremendous advances in scientific knowledge have been made to guide not only the better usage and processing technology of natural fibers but also the finishing technologies of textile fabrics [101]. Synthetic polymers with high performance and environmentally friendly cotton fabrics with softness, breathability and biodegradability are most commonly used in textiles and clothing industries. Textile products have been widely used in many fields, including health safety and protection, medical and hygienic, clothing, construction, agriculture, transport, electronics, geo-textiles and packaging and containment [102]. In the last few decades, surface treatment and functionalization of textile fabrics is a major focus of scientists and technologists. Cotton fabric is composed of natural cellulose polymer chains which have hydroxyl groups (OH) on their surfaces which impart hydrophilic, lyophilic and omniphilic properties to the cotton fabrics. These properties of the fabric render it vulnerable to contamination by biological and chemical agents and reduce its applications in many fields. To make textile fabric water repellent and resistant to organic contaminants, cotton fabric has been coated with hydrophobic agents. However, when cotton fabric is treated with some reagents it loses its inherent properties such as breathability, softness and mechanical properties. Hence, it is of great importance to create superhydrophobic textiles which retain their inherent properties. In this section, development of superhydrophobic textiles with their applications in various fields will be discussed.

### 6.1. Robust Superhydrophobic Textiles

As discussed in the previous sections, the superhydrophobicity of surfaces is attributed to two major parameters: (1) the presence of nano or micro hierarchical topographies and re-entrant structures on the surface of substrate; (2) the attachment or coating of low surface energy materials on the surface of substrate. Textile fabrics have inherently hierarchical structures on the surface. Hierarchy of the textile fabrics varies according to the nature and pattern of weaving and knitting processes which play a major role to develop superhydrophobic textiles. However, the polar groups (OH) present on the surface of textile fabric make it highly hydrophilic. To make it hydrophobic, the surface chemistry of textile fabrics needs to be changed either by the chemical modification of the surface or by attaching hydrophobic material on the surface. Many techniques have been used to develop superhydrophobic surfaces on the textile fabrics.

#### 6.1.1. Sol–Gel Method

This process is commonly known to be universal due to its flexibility with regard to productive capacity and coating technology. It has many advantages over other techniques used for the functionalization of textile fabrics due to following reasons [103]:

- Silica, modified silica and other metal oxide nano-sols with particle diameters ranging from 1 to 100 nm can make transparent and well adhered oxide layers on textiles.
- The oxide layers formed impart and enhance mechanical properties to the fabrics, e.g., wear and abrasion resistance.
- The metal oxide layers formed offer further possible ways to modify and enhance the surface functionality of the fabrics.
- The oxide layers formed by this method are stable against heat, light, microbial and chemical attacks.
- The oxide layers formed can carry other functional additives such as inorganic particles and polymers. It is easy to control the pore size, porosity level and immobilization of imbedded compounds.
- This process can be performed in conventional coating systems such as pad-dry-cure and simple dip-coating methods under normal temperature and pressure conditions.

This method consists of three basic steps: First, nano-particulate sols are prepared by acid or base catalyzed hydrolysis of corresponding precursors, including metal or silicon alkoxides in aqueous media or other water miscible solvents (e.g., the Stober method). Organic solvents, such as alcohols, are mostly used because they provide high storage stability, excellent adherence on textile fabrics and easy evaporation of the solvent at low temperature. Second, these nano-sols are applied on the textile substrates using the simple pad-dry-cure or dip-coating method. In this step, the silica, metal oxide or other nano-particles are condensed to form a lyogel layer on the substrate surface. Third, the solvent is evaporated resulting in a xerogel layer with a rough structure [103].

Final properties, such as density, porosity, roughness, critical thickness and mechanical properties can be controlled by governing the condensation and drying parameters of the sol–gel method. The nano-sols obtained by acid hydrolysis result in dense layers with weak cross-linked products in the condensation step whereas the nano-sols obtained by alkali catalysis form nano-particle layers with larger pores. In order to enhance the surface roughness and water repellency, some hydrophobic additives with long alkylsilane chains, alkyl groups (R) or perfluoroalkyl groups are introduced in the metal oxide nano-layers by chemical modifications. Many studies have been conducted to develop superhydrophobic textiles using the sol–gel method for perspective practical applications, e.g., rain clothes and tents and oil repellent fabrics [104,105,106].

#### 6.1.2. Admicellar Polymerization Technique

The admicellar polymerization process is another field of surface functionalization of fabrics. The working principle of this technique is a surface analogous to the emulsion polymerization process. It mainly consists of two steps: Firstly, surfactant is adsorbed on the substrate surface to form a bilayered aggregate of monomer at the substrate/solution interface. After a specific level of adsorption (critical admicelle concentration), the surfactant starts changing in morphology and orientation. This process of re-orientation allows the monomer to concentrate in the surfactant, termed as adsolubilization [107,108]. Secondly, in-situ polymerization starts by adding a suitable initiator in the admicelle and thin films are formed on the substrate surface to generate required morphology and functionality depending on the nature of the monomers. Many polymeric thin films have been applied on different solid substrates using this method. Recently, this approach has been applied on textile substrates to develop flame retardant, hydrophobic and UV-absorbing fabrics [109,110,111,112,113]. A rear research group developed a stain resistant cotton fabric by using admicellar polymerization. They used partially fluorinated alkyl acrylates as monomers in the presence of fluorosurfactants and a acrylamide bonding agent resulting in durable stain resistant finishes on the knitted cotton fabric [113,114].

#### 6.1.3. Direct Fluorination Modification

Modification of the substrate by introducing some fluoro groups on the surface lowers its surface energy. The lowering of surface energy induces hydrophobic properties in the substrate. One of the commonly used methods to introduce fluoro groups on the textile surface is direct fluorination. The direct fluorination is carried out in the presence of fluorine gas or plasma and the fluorine atoms attach covalently with the surface [115]. The main advantages of this method are: (1) In this process, only gases are used and it is said to be a dry process and needs no extra catalyst or initiator; (2) This process is carried out at room temperature or even low temperature; And (3) only a thin layer of the substrate is modified and bulk properties of the material remain unchanged. However, this modification process is highly exothermic and there is pressure of the fluorine gas in the reaction gaseous mixture renders the reaction dangerous thus requires careful handling of the reaction. [110].

#### 6.1.4. Electrospinning 

Electrospinning is one of the promising techniques applied for the production of superhydrophobic textiles. This system is easy to operate and offers many controllable parameters in order to obtain the required surface geometries. It can be used to obtain very similar fibers to those that are found in the natural hydrophobic systems. The electrospinning system basically consists of three main parts: a feeding system that consists of a container with precursor sols connected with a metallic needle and a pump, a high-voltage power supply to which a needle is connected that creates the charge on the precursor droplet and a collector on which the resulting nano fibers are deposited. The precursor sol can be of polymer solutions, melt or sol-gels etc. The pump injects the precursor at a constant rate. The electrical field works against the surface tension of the precursor solution and the droplet is converted to a conical shape called a Taylor cone and when electrical force is increased to an extent where it overcomes the surface tension, a thin jet of the precursor ejects from the Taylor cone tip. The jet finally reaches the collector and makes a non-woven mat of fine fibers. A wide range of materials can be used to obtain a variety of micro and nano fibers using this approach. In addition to the raw material selected, the processing parameters play a critical role in the final properties of electrospun fibers. The diameter, morphology, and fiber arrangements on the substrate surface greatly depend on the processing conditions. In order to create superhydrophobic surfaces by using this technique, beaded and nano-particles are preferred [116,117]. In this section, few parameters related to the raw material selection, processing conditions and system design are summarized in the Table 2. However, detailed effects of these parameters can be found in some other articles [118].

#### 6.1.5. Polymerization

Graft polymerization of hydrophobic monomers on the surface of textile fabric is the most effective method to achieve the robust superhydrophobic textiles. In this method, one end of the polymer makes covalent bonds with the textile fabric thus enhancing the durability and abrasion strength of the fabric, while the other end of the polymer renders the substrate textile fabric superhydrophobic. Organo-silanes [119,120,121,122], metal oxide with some hydrophobic materials [123,124,125,126,127,128], polymers [129,130,131,132,133,134], metals [135,136] and polymer composite materials [137,138] have been used on textile surfaces to achieve robust superhydrophobicity [139].

#### 6.1.6. Initiated Chemical Vapor Deposition (iCVD) and Atom Transfer Radical Polymerization (ATRP)

**Figure 10:**
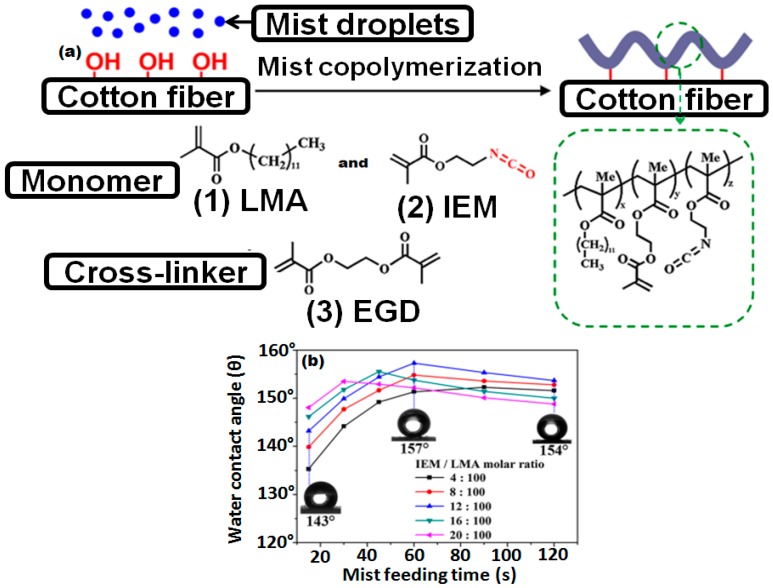
(**a**) Mechanism of mist copolymerization on the cotton fiber surface; (**b**) Variation of WCA values of modified surfaces with the mist feeding times. Reprinted from [129], Copyright Springer Science, 2015.

Initiated chemical vapor deposition (iCVD) and atom transfer radical polymerization (ATRP) techniques have been used to grow hydrophobic polymer chains on the textile surface. The iCVD process is a solvent free single step process used for the deposition of polymer chains on the surface of a variety of substrates. In this process, the monomer and an initiator are vaporized in a closed chamber. Vaporized initiator molecules, upon contact with a hot filament, generate free radicals by thermal decomposition. These free radicals strike with the monomer molecules and activate the free radical polymer chain reaction. As a result, the polymer films are deposited on the substrate at mild temperature (15–40 °C). Im and coworkers used this technique to produce robust superhydrophobic polyester fabrics with high mechanical and chemical stability. They deposited a first layer of poly(1,3,5,7-tetravinyl-1,3,5,7-tetramethylcyclotetrasiloxane) and a second layer of poly(1H,1H,2H,2Hperfluorodecylacrylate) [138]. A similar technique termed as mist polymerization has been used to create superhydrophobic cotton fabrics by grafting lauryl methacrylate using ethylene glycol dimethacrylate as a crosslinker. Mist polymerization is a two step process. In the first step, the substrate surface is activated by some chemical agent and in the second step the vaporized monomer is grafted on the surface [130]. In their next study, Wang and coworkers modified the mist polymerization process by mixing the initiator and different kinds of monomers in one pot. Later, that mixture was atomized on the cleaned cotton fabric. The block co-polymerization phenomenon was observed in which three monomers had undergone co-polymerization on the surface of the cotton fabric as shown in the schematic diagram in the Figure 10a. These block co-polymers imparted superhydrophobic properties to the fabric and a high contact angle (>151°) was obtained. The surface morphology largely depends on the polymerization conditions, concentration of the monomers and length of polymer blocks on the of substrate surface [129].

**Figure 11:**
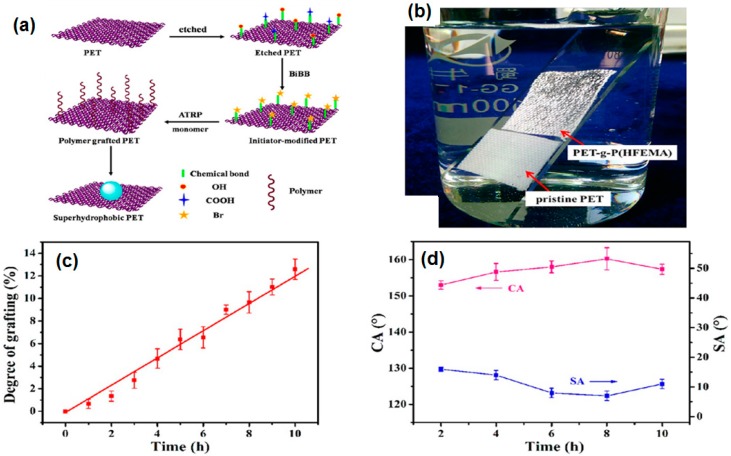
(**a**) Schematic illustration of polymer grafting on the substrate surface; (**b**) total light reflection on the polymers grafted surface showing robustness; (**c**) DG increase with time of polymerization; and (**d**) water contact angle and sliding angle with increasing time of polymerization. Reprinted from [128], 2015 Copyrights American Chemical Society.

Atom transfer radical polymerization (ATRP) is the most widely used “grafting from” in which polymer chains grow from the surface of substrates. A controlled rate of polymerization with specific site architecture on the surface of the substrate, versatility and compatibility for a large variety of solvents and monomers make the ATRP the most suitable synthetic technique to graft superhydrophobic moieties on the surface of substrates [140]. The ATRP process is carried out in three steps. In the first step, the substrate surface is washed and prepared by etching for grafting of polymers. Then ATRP initiator is applied on the substrate surface. In the last step, a polymer of desired properties is grown on the surface of the activated substrate surface. Polymerization conditions i.e., reaction time and monomer flow rate are the key parameters to control the degree of polymerization. The degree of polymerization may be defined as the number of monomer units that have been polymerization into the polymer chain. Superhydrophobicity of the substrate surface is controlled by the degree of polymerization or degree of grafting from the substrate surface. The degree of grafting (DG) is calculated from the weight increase of the substrate using this equation:

DG (%) = (W_1_ − W_0_)/W_0_ × 100 (12)

where W_0_ and W_1_ are the weight of substrate before and after grafting. The Jia research group grafted fluorinated methacrylates on poly(ethylene terephthalate) fabrics [128]. The degree of grafting increased linearly with time but surface roughness and superhydrophobicity first increased with an increasing degree of grafting, however, after some time, with an increasing degree of grafting, the roughness decreased which resulted in a decrease in the superhydrophobicity as shown in the Figure 11c,d, respectively. The polymer grafted fabric shows robust superhydrophobicity even after 100 laundering cycles, 2500 abrasion cycles and exposure to UV irradiation as shown in Figure 11b.

### 6.2. Superhydrophobic Textiles for Oil–Water Separations

**Figure 12:**
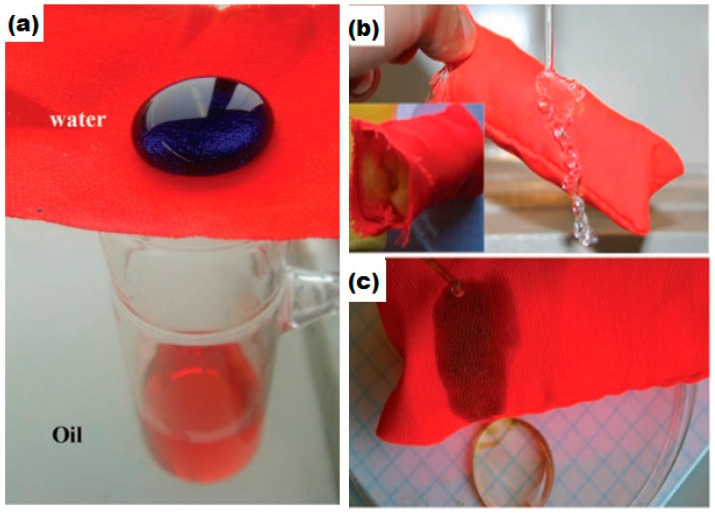
(**a**) Experimental setup for oil–water separation; (**b**) water flow on coated surface; and (**c**) selective absorption of crude oil from water Reprinted from [149]. Copyrights 2011 Wiley-VCH Verlag GmbH & Co. KGaA, Weinheim, Germany.

Environmental and water pollution due to chemical and oil leakage caused some of the most serious and harmful incidents in the world since the last century. Owing to these incidents, there is a huge demand for the development of functional materials for the control and removal of harmful contaminants from the environment such as oil–water separation and the removal of toxic metals from water [141,142]. Much advancement has been made in this regard and functional membranes have been developed via self assembly, electro spinning, selective etching and lithographic techniques [143,144,145]. However, the materials and methodologies used have limitations for the production of such materials on an industrial scale because of the complex fabrication procedures, low flexibility, poor stability, high cost and poor recyclability. Therefore, it is of great importance to develop simple, cost effective and environmentally friendly membrane materials to overcome the above mentioned problems. The development of materials for the separation of oil from aqueous media requires specific surface functionality and selective wettablity to liquids with different surface tensions. Superhydrophobicity and superoleophilicity of the membrane surface are required for the most efficient oil–water separations [146,147,148,149]. Selective wettability of solid surfaces is achieved by controlling the surface chemistry and topography. Creating surface roughness by the introduction of controlled nano and micro architecture on the solid surface supports the entrapment of an air cushion on the solid surface which, in turn, makes the hydrophobic surface superhydrophobic and oleophilic surface superoleophilic [132,150,151]. Superhydrophobic surfaces that have been used for oil–water separation include metal meshes [152,153,154], polymer composites [155,156], metal oxides [157] and filter papers [158]. However, these materials face some limitations for large scale applications due to potential toxicity, poor mechanical stability and low flexibility. Textile based superhydrophobic materials for practical applications in the field of oil–water separation have received rigorous attention owing to their high absorption ability, flexibility, low density, high mechanical stability and low cost under harsh environmental conditions. Several studies have been conducted to develop robust superhydrophobic textile fabrics for the separation of oils from aqueous media. The Seeger research group has developed superhydrophobic fabric by chemical vapor deposition (CVD) of silicon nanofilaments on a polyester substrate. Their study shows a good level of oil absorption from aqueous media repelling the water but poor oil recovery from the fabric and no recyclability was evaluated, as shown in the Figure 12 [149].

In another study, stearic acid modified ZnO/polystyrene nanoparticles were coated on the cotton fabric by the simple drop coating method for the separation of oil from water. Oil passed through the ZnO/polystyrene coated cotton fabric while the fabric showed high water repellency with a water contact angle of 153°–155° [159]. Oil–water separation efficiency can be calculated from the ratio of weight of water collected after separation to the weight of water initially added.

**Figure 13:**
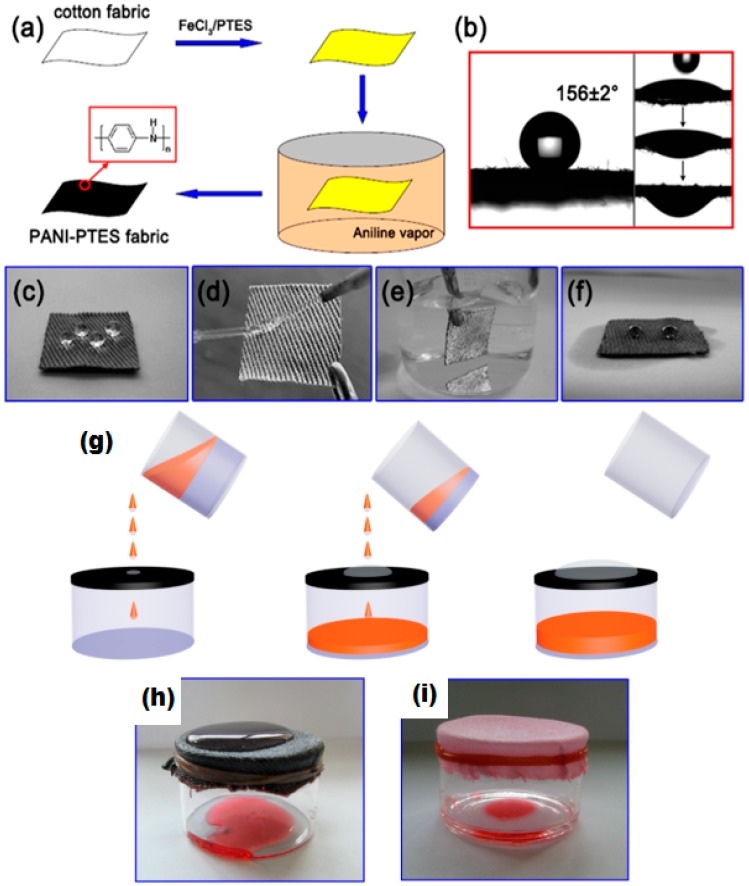
(**a**) Schematic illustration of vapor phase deposition of PAN on cotton fabrics; (**b**) Water droplet and hexadecane droplet on the superhydrophobic cotton fabric; (**c**) Water droplets on the coated textile; (**d**) water bouncing off the surface; (**e**) coated fabric immersed in water; (**f**) water droplets on the oil contaminated textile; (**g**) schematic illustration of the oil–water separation process; (**h**) hexadecane–water mixture on the superhydrophobic fabric; and (**i**) hexadecane–water mixture on raw fabric. Reprinted from [161]. Copyrights 2013 American Chemical Society.

Oil–water separation efficiency (η) = (W_2_/W_1_) × 100 (13)

where W_1_ and W_2_ are the weight of water initially added and the weight of water after separation respectively. Oil–water efficiency was evaluated at different weight concentrations of modified ZnO/polystyrene in THF. Coating with 2.22 wt % of ZnO/polystyrene in THF had oil–water efficiency of 92%. Cotton fabric coated with SiO_2_ nanoparticles along with octadecyltrichlorosilane (OTS) by the sol gel method shows 50 times greater absorption of oil from water than its own weight. A total of 90% of oil was recovered from the coated fabric by vacuum filtration [160]. Zhou et al. developed a robust superhydrophobic/superoleophilic cotton fabric for oil–water separation by using in-situ vapor phase deposition of fluorinated alkyle silane and polyaniline (PAN) [161]. The coated cotton has high stability under harsh conditions of temperature, acid and bases. A high water contact angle of 156° and 97.8% oil–water efficiency was obtained. Moreover, the fabric has high recyclability. After 30 cycles, the water contact angle was decreased to 140° while oil–water efficiency of 94% was observed. In this study, hexadecane with low surface tension (γlv = 27.5 mN/m) liquid was used. Surface tension of the coated fabric lies between water and hexadecane which renders it superoleophilic, showing permeability and wettability to hexadecane. Additionally, a mirror-like bright and reflective surface underneath the water droplet, as shown in Figure 13c,e confirms the presence of an entrapped air cushion, thus stabilizing the Cassie–Baxter state.

**Figure 14:**
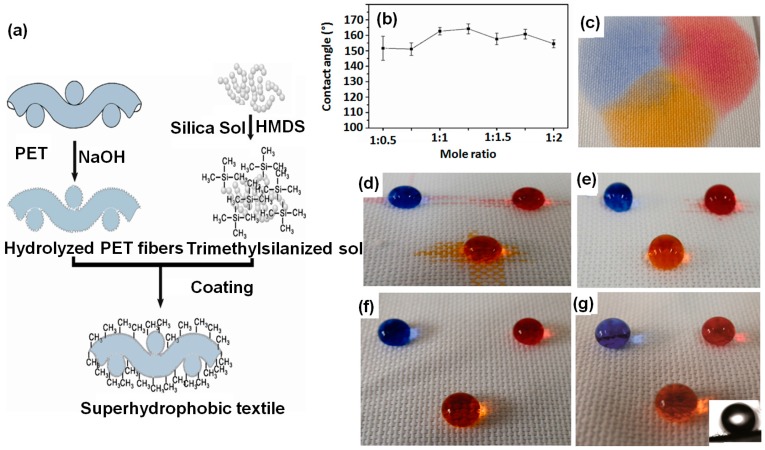
(**a**) Schematic illustration of –Si(CH_3_)_3_ functionalized SiO_2_ nanoparticles fabrication on poly(ethylene terephthalate) (PET) fabric; (**b**) change in CA with monomer ratio; (**c**) original PET fabric; (**d**) coated fabric with TEOS:HMDS molar ratio of 1:0.5; (**e**) 1:0.75; (**f**) 1:1.25; and (**g**) 1:1.75; inset in (**g**) shows the rolling state of water drop. Reprinted from [127]. Copyrights 2013 Elsevier B.V.

The Jia research group decorated –Si(CH_3_)_3_ functionalized SiO_2_ nanoparticles on poly(ethylene terephthalate) (PET) fabric to achieve syperhydrophobic/superoleophilic surface morphology [127]. The schematic illustration of the sol–gel process is given in the Figure 14a.

**Figure 15:**
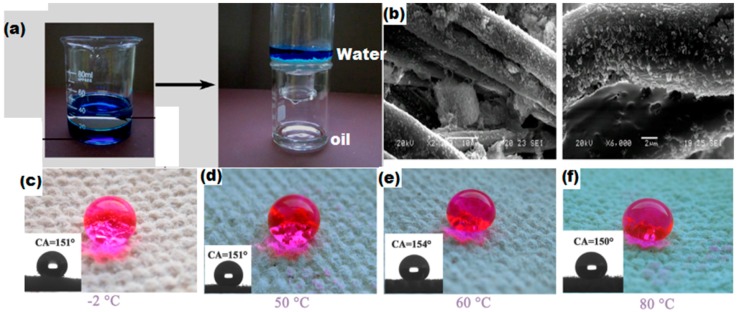
(**a**) simple oil–water separation setup; (**b**) SEM images of fabrics after being treated with SiO_2_/PS suspensions; (**c**–**f**) Water droplet at different temperatures standing on the fabric coated with SiO_2_/PS. Insets in the lower left-hand corner of each panel are images of the static water droplets (4 µL). Reprinted from [126]. Copyrights 2013 Elsevier B.V.

The development of superhydrophobic surfaces requires micro/nano level surface roughness and low surface energy. Polystyrene modified SiO_2_ nanoparticles, when deposited on the probe surface, create micro/nano level roughness and lower the surface energy, thus making the surface highly water repellent. The Zhang research group used a polystyrene/SiO_2_ nano-composite coating on the textile fabric (70% cotton and 30% polyester) to create a superhydrophobic/superoleophilic surface for oil–water separation. They reported oil–water separation efficiency of 93%–98.7%. Flexible and thermally stable superhydrophobic fabric was obtained as shown in the Figure 15 [126].

### 6.3. Superhydrophobic Textiles for Ultraviolet Radiation Shielding

The ozone layer has been protecting life on Earth for hundreds of years by absorbing harmful radiations coming from sun. The major part of harmful radiations coming from the sun consists of ultraviolet (UV) radiations. UV radiations have been divided into three categories depending on the wavelength i.e., ultraviolet-A (UV-A), ultraviolet-B (UV-B) and ultraviolet-C (UV-C). Wavelengths of UV-A, UV-B and UV-C range, 315–400 nm, 280–215 nm and 200–280 nm respectively. The most dangerous for life on Earth is the UV-B region, which is absorbed by the ozone layer. However, the ozone layer has been depleted by many chemicals, including halocarbons and green house gases released by human activities. This ozone depletion allows the UV-B radiation to pass through and affect human life [162]. The UV-B radiation has deteriorating effects on human health, causing skin damage such as premature skin aging, allergies, sunburn and even increasing the rate of skin cancer [163,164,165].

**Figure 16:**
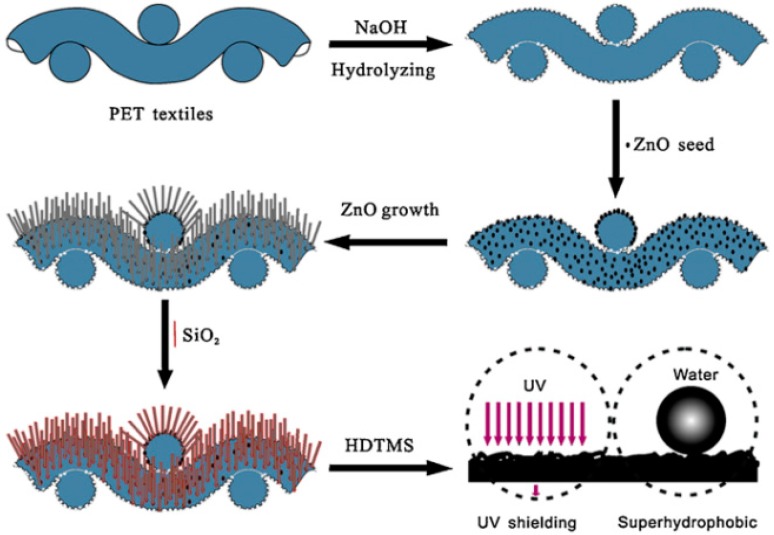
Schematic illustration of superhydrophobic PET fibers fabrication. Reprinted from [173]. Copyrights 2013 Elsevier B.V.

UV blocking products such as cosmetics have been used to protect human skin from UV radiations. However, it is of high demand to develop UV blocking textile products as most parts of the body are covered by textile clothing. Various studies have been conducted to prepare UV blocking fabric using white pigments [165,166,167,168,169,170,171,172]. Moreover, innovations in green technologies, development of multifunctional textile fabrics with self-cleaning properties, superhydrophobicity, antibacterial activity and UV blocking ability are emerging research fields in textiles and clothing industries. The Jia research group observed that when PET fabric is coated with ZnO/SiO_2_ pencil-like rods with diverse diameters, it shows absorption of UV radiations. This absorption of UV radiation results from the quantum confinement effect. This effect can be explained as follows: when the size of nano-particles decreases to a certain point where its radius becomes comparable to the wavelength of an electron, the electronic structure of the particles becomes discrete in defined energy levels. This confinement leads to the efficient separation of electron whole pairs and absorption occurs in the UV region. Also, light scattering from the ZnO nano-particles coating on the surface of the fabric results in the UV radiation shielding. Moreover, this pencil-like rods coating of ZnO/SiO_2_ also created superhydrophobicity with a water contact angle of 160° ± 5° as shown in Figure 16 [173]. However, the water contact angle decreases to 90° when exposed to UV light as hydrophilic groups appear on the surface due to UV radiation absorption.

The coating of CeO_2_ nanoparticles also imparts superhydrophobic and UV protection properties to the cotton fabric. The band gap energy in the UV region and large refractive index of CeO_2_ result in UV absorption and UV scattering on the surface, respectively. Nano/micro level valleys and hills, such as roughness created on the surface of cotton fabric by dip-pad-dry coating of CeO_2_ nanoparticles, results in the superhydrophobic surface with a static water contact angle of 158° [174]. Layered double hydroxides (LDHs) have also been observed to exhibit the UV blocking and superhydrophobic characteristics when coated on textile fabrics. LDHs are inorganic materials with the general formula [M^2+^_1−x_M^3+^_x_(OH)_2_]^An−^_x/n_·mH_2_O, which form layer-by-layer assembly of hydrated anions and positively charged hydroxide layers. M^2+^ and M^3+^ are metal ions which occupy octahedral sites in the layer-by-layer crystalline structure of the host layer and A^n−^ is the anion present between the layers. In the layer-by-layer structure, trivalent metal ions are partially substituted by bivalent metal ions which result in the appearance of positive charge on the hydroxide layer. The Lin research group used an organic based UV absorber intercalated into the LDH nano layers. This electrostatic layer-by-layer (LBL) assembly imparted dual-functions of UV-blocking and superhydrophobicity to the substrate textile fabrics [175].

### 6.4. Flame Retardant Superhydrophobic Textiles

Fire accidents occurring every year in the whole world cause a serious loss of human lives and properties. Fire risk is the potential of materials to ignite and contribute to fire propagation. All materials containing carbon are flammable and combust when heat and oxygen are present. Cotton, most commonly used as textiles, is highly flammable. The potential of a material to ignite with increase a fire is measured in terms of flammability. Measurement of the limiting oxygen index (LOI) is one of the best and most commonly used methods to evaluate the flammability of materials. The limiting oxygen index (LOI) can be defined as “the minimum amount of oxygen gas required for the ignition of a material in an oxygen–nitrogen mixture”. The lower the value of LOI, the higher the flammability of materials. Cotton has the LOI value of 18%–19% which renders it highly flammable in the presence of heat, as oxygen is present in a sufficient amount in the air. The large scale applications of cotton fabrics in daily life thus require cotton fabric to be flame retardant to reduce the fire risk. Flame retardant materials are those materials which have low fire risk and are resistant to ignition. Flame retardant materials reduce fire risk and lower the heat release rate during combustion in three different ways: (1) Gas phase flame retardancy; in this mechanism the flame retardant material reduces the heat release in the gas phase by scavenging the free radicals that propagate ignition. These flame retardant materials consist of halogen and phosphorus based additives; (2) Endothermic flame retardancy; this mode of flame retardancy is based on metal hydroxides and carbonates as a flame retardant additive. These additives undergo endothermic degradation, thus reducing the heat responsible for ignition and also producing non-flammable gases such as CO_2_ and water vapors that dilute the fuel gases resulting in low fire risk; (3) Char formation; in this mechanism, the flame retardant additives form a layer of non-pyrolyzable and heat insulator char on the surface of the substrate to reduce the heat release and thus prevent fire propagation. These flame retardant additives mostly consist of intumescents and nano-composite materials [176].

The development of flame retardant textiles with superhydrophobicity has been reported in various studies. Char formation by intumescent and nano-composite materials is the most common way to prevent the textile products from fire risk. The Sun research group has used layer by layer assembly of branched poly(ethylenimine) (bPEI), ammonium polyphosphate (APP), and fluorinated-decyl polyhedral oligomeric silsesquioxane (F-POSS) to create multiple functionality on the cotton fabric such as superhydrophobicity and flame retardancy. They have reported that, on exposure to fire, cotton cellulose and bPEI undergo dehydration catalyzed by APP and produce a heat insulating char layer on the cotton surface and the gases produced in decomposition of bPEI generate the porosity in the char layer. This porous char layer imparts flame retardant properties to the cotton fabric. Superhydrophobicity of the fabric was reported as a result of the F-POSS layer on the fabric. The LOI value of the coated fabric was increased to 20% [177].

**Figure 17:**
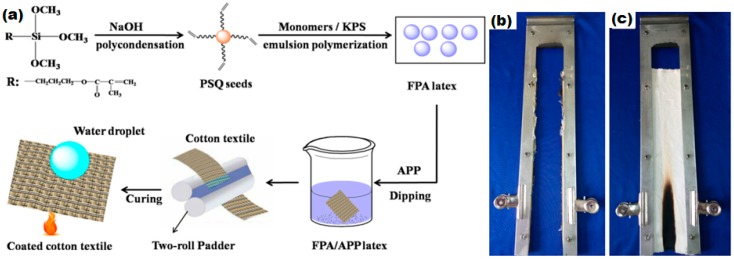
(**a**) The schematic illustration of the coating of a flame retardant additive on the cotton; (**b**) digital image of pristine cotton; and (**c**) image of flame retardant coated cotton fabric. Reprinted from [179]. Copyrights Springer Science + Business Media Dordrecht 2016.

ZnO nano-particles and 3-aminopropyl(diethoxy)methylsilane/ZnO nano composites have also been reported to impart flame retardant and superhydrophobic properties to the cotton fabric. The flame retardant properties are resulted from the heat insulating barrier developed on the cotton surface by the nano-composite layer and the roughness induced by the ZnO nano-layer resulted in superhydrophobicity with a water contact angle of 152 ± 2 [125,178]. In another study, it has also been reported that branched poly(ethylenimine), Polymethacryloxypropylsilsesquioxane (PSQ) latex particles and ammonium polyphosphate produce a char layer on the surface of cotton fabric on burning, which further reduces the fire propagation and the LOI value was increased to 24% [179]. The schematic illustration of the coating of flame retardant particles on the cotton fabrics is shown in Figure 17a.

## 7. Conclusions and Future Work

In this review article, a concise and emphasized study has been conducted to explain the fundamental principles, mechanisms, past experimental approaches and ongoing research in the development of superhydrophobic textiles. Various theories have been developed to explain the mechanism of the wetting process and the creation of superhydrophobic surfaces. Young’s equation, and Wenzel and Cassie–Baxter models are the most important and fundamental theories to explain superhydrophobicity. According to these theories, the substrates with a water contact angle of greater than 150° and a water droplet sliding angle of lower than 10° are defined as superhydrophobic. The superhydrophobicity of a surface depends on its surface geometry, surface chemistry and surface energy. Development of hierarchical re-entrant topographies and coating of the surfaces with low surface energy materials are the major factors to develop superhydrophobic surfaces. Textile fabrics have inherently hierarchical structures on the surface of fibers which play a major role in the development of superhydrophobicity. However, in this review, some major study gaps have been found that are not addressed clearly: (1) Hierarchy of the textile fabrics varies according to the nature and pattern of the weaving and kitting processes. In the weaving process, various weave structures are followed to develop plain, twill, satin and sateen fabrics. While, in the knitting process, single jersey, double jersey, interlock, rib, tricot and pearl strucutre based fabrics have been developed and used on industrial scales. When persuing the studies at nano-scale level, these structures may play a major role in surface geometry analysis. It has been observed in this review that no systematic study has been conducted to evaluate the effect of these parameters in surface topographies analysis; (2) Superhydrophobic textiles for ultraviolet shielding have been developed, however the sun protection factor (SPF) and standard UV protection analysis parameter have not been evaluated for the UV shielding superhydrophobic textiles. It is suggested that these study gaps should be explored in analysing the surface topographies at nano-level and UV shielding evaluation of the superhydrophobic textile fabrics.

## Figures and Tables

**Table 1:** Physical properties of natural superhydrophobic systems. Data adopted from ref. [93,95].

Natural Surface	Surface Structure	Contact Angle (θ)	Properties
**Lotus leaf**	Hierarchical, wax tubules	160°	Superhydrophobic Low drag Low adhesion
**Rice leaf**	Sinusoidal grooves covered with micropapilla and nanobumps	164°	Superhydrophobic Low drag Low adhesion
**Butterfly wing**	Shingle-like scales with aligned microgrooves	161°	Superhydrophobic Low drag Low adhesion
**Fish scale**	Overlapping hinged scales	58°	Mucus Hydrophilic Low adhesion
**Shark skin**	Overlapping dermal denticles with triangular riblets	n/a	Mucus Hydrophilic Low adhesion

**Table 2:** Electrospinning parameters governing the final properties of obtained fibers [117].

Material Properties	Processing Conditions	System Design	Ambient Conditions
Surface tension	Electric field strength	Design of the needle	Temperature
Viscosity	Type of electrical signal	Geometry of the collector	Humidity
Conductivity	Solution charge polarity		Air velocity
Polymer type	Tip to collector distance		Medium of the system
Polymer MW	Flow rate		
